# Factors associated with patient‐physician discordance in a prospective cohort of patients with psoriatic arthritis: An Asian perspective

**DOI:** 10.1111/1756-185X.13568

**Published:** 2019-04-03

**Authors:** Charmaine Tze May Wang, Yu Heng Kwan, Warren Fong, Shu Qin Xiong, Ying Ying Leung

**Affiliations:** ^1^ Department of Rheumatology & Immunology Singapore General Hospital Singapore City Singapore; ^2^ Duke‐NUS Medical School Singapore City Singapore

**Keywords:** discordance, patient global assessment, patient‐reported outcomes, psoriatic arthritis

## Abstract

**Objectives:**

To evaluate factors associated with patient‐physician discordance in a multiethnic Asian cohort of psoriatic arthritis (PsA) patients.

**Methods:**

We used data from a prospective cohort of consecutive patients with PsA fulfilling the Classification Criteria for Psoriatic Arthritis, recruited from a single center in Singapore. Sociodemographic, clinical data and patient‐reported outcomes were collected using a standardized protocol at baseline, 4 months, 8 months, 1 year, 2 years and 5 years. patient‐physician discordance was defined as patient global assessment minus physician global assessment (PGA‐PhGA). We evaluated variables associated with patient‐physician discordance using generalized linear regression to control for within‐subject effect.

**Results:**

One hundred and fortytwo patients (51.4% male, 66.2% Chinese, mean [SD] age and duration of illness 51.1 [13.8] years and 27.5 [98.3] months) were recruited at baseline. Paired results for PGA and PhGA were available for 291 visits with median (interquartile range) follow‐up time of 11.6 (17) months. In univariable analysis, duration of illness, fatigue, pain, tender and swollen joint count, dactylitis count, and health‐related quality of life (Short Form‐36) domains were significantly correlated with patient‐physician discordance. In multivariable analysis, age, fatigue level, pain score were positively associated with patient‐physician discordance, while swollen joint count and mental health were negatively associated with patient physician discordance.

**Conclusions:**

Increased age, higher fatigue levels, higher pain score  and poorer mental health may explain underestimation of disease activity by physicians. Physicians’ overestimation of disease activity may be explained by higher swollen joint counts.

## 1 INTRODUCTION

Psoriatic arthritis (PsA) is a chronic inflammatory articular disease with diverse clinical manifestations.^1^ Destructive changes in bones can develop early in the disease, resulting in joint damage and loss of function.^2^ Furthermore, higher inflammatory burden over time may lead to atherosclerosis and increased cardiovascular morbidity and mortality.^3^, ^4^ Hence appropriate assessment and management of PsA, is vital to ensure preservation of joint structure, improvement of function, quality of life and reduction in mortality.^5^ The lack of available biomarkers to assess disease activity in PsA emphasizes the importance of shared decision‐making between patient and physician to ensure optimal treatment outcomes.^6^, ^7^ However, the patient's assessment of their own disease activity may not always be similar to the physician's assessment of disease activity. This lack of agreement between patient and physician will result in discordance.^8^, ^9^ It has been shown that patient‐physician discordance can result in patient dissatisfaction, poor compliance to treatment and increased healthcare costs in spondyloarthritis.^10^ Hence understanding factors associated with patient and physician discordance is important to facilitate shared decision‐making and optimal treatment management.

There are studies, mainly in Caucasian populations, that have reported factors such as fatigue, pain score, disability and tender and swollen joint counts to be associated with patient‐physician discordance.^11^, ^12^ However, none of these studies exist in the Asian population. Environmental and ethnic differences have been reported to affect clinical manifestations and disease progression among Asian patients with PsA.^14^, ^15^ There is clear evidence that perceptions of health and illness differ in different socio‐cultural contexts. Studies have shown that Caucasians have more biological and psychological beliefs, while Asians have sociological and theological explanations for health.^17^, ^18^ People from different ethnic groups also have differences in perception and reporting of pain.^19^ Singapore is a multi‐ethnic Asian country with Chinese (74.1%), Malays (13.4%), Indians (9.2%), and others (3.3%).^20^ It would be an ideal setting to evaluate factors related to discordance between patients and physicians regarding PsA in Asians.

The objective of this study was to evaluate factors associated with the discordance in patient global assessment (PGA) and physician global assessment (PhGA) in patients with PsA in a multi‐ethnic Asian population in Singapore.

## 2 METHODS

### 2.1 Study population

We used data collected from the PRESPOND registry, which is a prospective cohort of consecutive patients with spondyloarthritis recruited in designated clinics in Singapore General Hospital from 2012.^21^ We included patients who fulfilled the Classification Criteria for Psoriatic Arthritis,^1^ and had complete data for PGA and PhGA. The study protocol was reviewed and approved by the SingHealth Centralized Institutional Review Board (CIRB Ref: 2012/498/E). Written informed consent was obtained from each patient prior to this study.

### 2.2 Data collection

Sociodemographic, clinical data and patient‐reported outcomes (PROMs) were collected using a standardized protocol during the following time points: baseline, 4 months, 8 months, 1 year, 3 years and 5 years. We collected sociodemographic data including age, gender, ethnicity (Chinese/Malay/Indian/others), education (none/primary/secondary/post‐secondary/tertiary) and duration of illness (PsA). Body weight and height without shoes were measured in all visits. Three designated physicians (YYL, WF, NLL) collected clinical data for swollen, tender, and damaged joint counts in a 66/68/68 diarthrodial joints diagram. Dactylitis count was assessed by counting the number of digits that are swollen and tender (0‐20); enthesitis was assessed using the 6‐point Leeds Enthesitis Index (LEI).^22^ Clinically damaged joints were assessed as previously described.^2^ In brief, these were joints that were ankylosed, loosened, subluxed; or had undergone surgeries due to PsA; or joints having a >20% decrease in range of movement. Psoriasis was assessed by using the Psoriasis Area and Severity Index (PASI).^23^

Patient‐reported outcomes included pain scores in the past week on a 0‐100 mm visual analogue scale (VAS) (0 no pain to 100 worst pain), Health Assessment Questionnaire Disability Index (HAQ‐DI) (0 with no disability to 3 severe disability),^24^ Medical Outcome Short Form‐36 (SF‐36) version 2 (eight domains with scores standardized 0‐100, higher scores indicate better quality of life).^25^ All PROMs were available in both English and Chinese, and were self‐administered by patients in the usual language they spoke. PROMs available in Chinese and English languages would cover 98% of Singaporeans.^20^

### 2.3 Patient global assessment and Physician global assessment

Patient global assessment (PGA) was evaluated using a 0‐100 mm VAS using this question: “What's your assessment of your disease activity during the last week?”.

Physician global assessment (PhGA) was evaluated by the designated physicians blinded to results of the PGA, using this question: “How would you rate the patient's joint disease activity?” It was rated on an 11‐point numeric rating scale (NRS) with 0 as no disease to 10 as severe disease.

For computation of patient‐physician discordance (PGA‐PhGA), first, PhGA in NRS was standardized to 0‐100. The PGA‐PhGA was calculated as PGA minus PhGA. Positive discordance is defined as PGA‐PhGA ≥20, indicating physicians underestimating patients’ perceptions of disease activity. Negative discordance is defined as PGA‐PhGA <−20, indicating physicians overestimating patients’ perceptions of disease activity.^11^, ^26^

### 2.4 Statistical analysis

Demographic and clinical characteristics were described. We compared discordance groups using descriptive parametric or nonparametric statistics as appropriate. In the univariable analysis, we presented the Spearman's Rho correlations for variables with both PGA and PhGA; and the discordance (PGA‐PhGA). Based on previous studies, we hypothesized that PGA would have strong correlations with pain and physical function but moderate correlation with joint count.^27^, ^28^ We also expected strong correlations to exist between PhGA and joint count. A Spearman's Rho >0.5 was considered strong, while 0.3‐0.49 was moderate and <0.3 was weak.^29^

In multivariable analysis, we evaluated variables associated with PGA‐PhGA using generalized linear regression to control for within‐subject effect. We considered variables that had *P* values <0.2 in the univariable analysis for further analysis. Variables measuring similar domains were collapsed (eg fatigue chosen instead of SF‐vitality; pain score chosen instead of SF‐bodily pain, swollen joint chosen instead of tender joint count, SF‐role‐physical instead of SF‐role‐emotional; all Spearman's Rho >0.70, *P* < 0.001). We also considered the following variables in the final model for theoretical importance or were shown to be important in other studies: age,^13^ gender,^11^ education,^30^ ethnicity,^14^ body mass index (BMI)^21^ and other PsA domains (skin and enthesitis).^13^ To exclude the effect of increasing numbers of follow‐up on PGA‐PhGA, we conducted sensitivity analysis limited to the baseline dataset.

All statistical hypothesis tests were 2‐tailed, and *P* values <0.05 were considered significant. Analyses were performed using the IBM SPSS Statistic Package, version 21 (IBM, Armonk, NY).

## 3 RESULTS

### 3.1 Patient characteristics

One hundred and forty‐two patients (51.4% male, 66.2% Chinese) recruited to the baseline protocol, who had completed results for PGA and PhGA were included in this study. Thirty‐eight percent of patients were classified having discordant PGA‐PhGA, of which 17.6% and 20.4% were positively and negatively discordant, respectively (Table 1). Patients with positive discordance had significantly higher scores for pain and fatigue severity. Patients with negative discordance had shorter duration of illness, significantly higher tender/ swollen joint counts. Chinese ethnicity had higher percentage of negative discordance, while non‐Chinese had higher percentage of positive discordance numerically. However, the differences were not statistically significant.

**Table 1:** Baseline characteristics of patients with psoriatic arthritis according to discordance categories (n = 142)

	Total (n = 142)	Negative discordance (n = 25)	Concordant (n = 88)	Positive discordance (n = 29)	*P* values
Age, y^a^	51.1 (13.8)	53.8 (14.4)	51.8 (13.2)	46.8 (14.7)	0.133
Gender, n (%)					
Female	69 (48.6)	14 (56.0)	42 (47.7)	13 (44.8)	
Male	71 (51.4)	11 (44.0)	46 (52.3)	16 (55.2)	0.691
Ethnicity, n (%)					
Chinese	94 (66.2)	20 (80.0)	58 (65.9)	16 (55.2)	
Non‐Chinese	48 (33.8)	5 (20.0)	30 (34.1)	13 (44.8)	0.157
Education, n (%)					
Secondary or below	85 (60.3)	15 (60.0)	55 (63.2)	15 (51.7)	
Post‐secondary	56 (39.7)	10 (40.0)	32 (36.8)	14 (48.3)	0.548
BMI, kg/m^2^ b	25.6 (6.7)	24.8 (4.3)	25.7 (6.3)	27.6 (8.6)	0.191
Duration of PsA, mo^b^	27.5 (98.3)	4.0 (44.0)	36.5 (102.8)	28.0 (100.0)	0.012
PGA (0100)^b^	31.0 (37.0)	8.0 (14.5)	30.0 (34.0)	51.0 (29.5)	<0.001
PhGA (0‐10)^b^	3.0 (2.25)	4.0 (2.0)	3.0 (2.0)	2.0 (2.0)	<0.001
Fatigue (0‐10)^b^	5.0 (4.0)	3.0 (4.0)	5.0 (4.0)	6.0 (3.0)	0.062
Pain (0‐100)^b^	27.0 (37.4)	18.0 (29.0)	27.0 (37.6)	46.0 (38.0)	0.031
Tender joint count (0‐66)^b^	1.5 (4.0)	3.0 (4.0)	2.0 (4.0)	1.0 (2.5)	0.020
Swollen joint count (0‐68)^b^	1.0 (3.0)	3.0 (6.0)	1.0 (3.0)	0.0 (1.5)	0.001
Clinically damaged joints (0‐68)^b^	1.0 (4.0)	2.0 (5.75)	1.0 (5.5)	0.0 (0.75)	0.008
Dactylitis count (0‐20)^b^	0.0 (1.0)	1.0 (2.5)	0.0 (1.0)	0.0 (0.0)	<0.001
LEI (0‐6)^b^	0.0 (1.0)	0.0 (1.0)	0.0 (0.0)	0.0 (1.0)	0.601
PASI (0‐72)^b^	1.5 (3.1)	1.6 (7.8)	1.3 (3.1)	1.7 (2.8)	0.783
HAQ‐DI (0‐3)^b^	0.25 (0.63)	0.25 (0.38)	0.25 (0.63)	0.25 (1.0)	0.641
SF36 subscales					
PF (0‐‐100)^b^	75.0 (40.0)	75.0 (35.0)	70.0 (40.0)	75.0 (60.0)	0.578
RP (0‐100)^b^	75.0 (50.0)	81.3 (25.0)	75.0 (81.4)	75.0 (43.8)	0.025
BP (0‐100)^b^	62.0 (33.0)	72.0 (38.0)	62.0 (33.0)	62.0 (32.0)	0.551
GH (0‐100)^b^	53.5 (27.0)	60.0 (22.5)	52.0 (30.8)	55.0 (25.0)	0.153
VT (0‐100)^b^	50.0 (21.9)	62.5 (21.9)	50.0 (28.1)	50.0 (25.0)	0.276
SF (0‐100)^b^	75.0 (37.5)	87.5 (25.0)	81.3 (50.0)	75.0 (12.5)	0.063
RE (0‐100)^b^	91.7 (50.0)	87.5 (25.0)	79.2 (50.0)	83.3 (25.0)	0.079
MH (0‐100)^b^	70.0 (63.8)	75.0 (22.5)	75.0 (30.0)	60.0 (25.0)	0.085

BMI, body mass index; HAQ‐DI, Health Assessment Questionnaire disability index; LEI, Leeds Enthesitis Index; PGA, patient global assessment; PhGA, physician global assessment; PASI, Psoriasis Area and Severity Index; SF‐36, Medical Outcome Short Form‐36; PF, physical function; RP, role‐physical; BP, bodily pain; GH, general health; VT, vitality; SF, social functioning; RE, role‐emotional; MH, mental health.

a Mean (standard deviation).

b Median (interquartile range).

From the prospective database with median (interquartile range) follow‐up duration of 11.6 (17) months, a total of 291 visits from the 142 patients had complete data for PGA‐PhGA for further analysis. The number of patients who reached certain follow‐up time points and who completed the protocol is shown in Table S1. The overall discordance rates in these 291 visits was 33.7% (12.4% positive and 21.3% negative discordance).

In the univariable analysis of the 291 visits from 142 patients, fatigue and pain were strongly correlated with PGA, while only moderately with PhGA. Tender and swollen joint counts were strongly correlated with PhGA but only moderately with PGA. This is consistent with our prior hypothesis. The correlations between quality of life SF‐36 domains and global assessments were moderate across domains, except that the correlation between SF‐bodily pain with global assessments was strong. (Table 2.). There were statistically significant correlations between PGA‐PhGA with fatigue, pain, tender/ swollen joint counts, dactylitis count, and SF‐36 domains.

**Table 2:** Spearman's Rho correlation of patient‐physician discordance with patient characteristics

	PGA	PhGA	PGA‐PhGA	
	Rho	Rho	Rho	*P* value
Age, y	−0.130*	−0.119*	0.012	0.832
BMI, kg/m^2^	0.077	0.021	0.012	0.844
Duration of PsA, mo	0.065	−0.160**	0.194	0.001
Fatigue (0‐10)	0.577**	0.369**	0.270	<0.001
Pain (0‐100)	0.750**	0.534**	0.333	<0.001
Tender joint count (0‐66)	0.370**	0.687**	−0.205	<0.001
Swollen joint count (0‐68)	0.264**	0.693**	−0.327	<0.001
Clinically damaged joint (0‐68)	0.067	0.198**	−0.092	0.119
Dactylitis count (0‐20)	0.166**	0.463**	−0.237	<0.001
LEI (0‐6)	0.251**	0.351**	−0.037	0.530
PASI (0‐72)	0.255**	0.332**	−0.016	0.790
HAQ (0‐3)	0.497**	0.504**	0.072	0.220
SF36 subscale				
PF (0‐100)	−0.397**	−0.377**	−0.083	0.160
RP (0‐100)	−0.433**	−0.412**	−0.097	0.098
BP (0‐100)	−0.575**	−0.509**	−0.152	0.009
GH (0‐100)	−0.488**	−0.409**	−0.136	0.020
VT (0‐100)	−0.468**	−0.408**	−0.113	0.055
SF (0‐100)	−0.483**	−0.354**	−0.197	0.001
RE (0‐100)	−0.418**	−0.362**	−0.115	0.049
MH (0‐100)	−0.421**	−0.277**	−0.157	0.007

BMI, body mass index; HAQ, Health Assessment Questionnaire for disability; LEI, Leeds Enthesitis Index; PGA, patient global assessment; PhGA, physician global assessment; PASI, Psoriasis Area and Severity Index; SF‐36, Medical Outcome Short Form‐36; PF, physical function; RP, role‐physical; BP, bodily pain; GH, general health; VT, vitality; SF, social functioning; RE, role‐emotional; MH, mental health.

*P < 0.05.

**P < 0.01 for significance values for correlations of global assessments with variables.

In the multi‐variable analysis, age, fatigue, pain were positively associated with PGA‐PhGA. In contrast, swollen joint count and poorer SF‐36 mental health were negatively associated with PGA‐PhGA. Ethnicity was not associated with PGA‐PhGA discordance (Table 3).

**Table 3:** Multivariable analysis for variables associated with patient‐physician discordance

Factors	β	95% confidence interval	*P* value
Age	0.270	0.040; 0.499	0.021
Gender			
Female	2.709	−2.117; 7.536	0.271
Male	Ref		
Ethnicity			
Chinese	−1.584	−6.673; 3.506	0.542
Non‐Chinese	Ref		
Education			
Secondary or below	−2.347	−7.970; 3.277	0.413
Post‐secondary (>10 y)	Ref		
Duration of illness	0.035	−0.001; 0.070	0.058
BMI	−0.388	−0.914; 0.139	0.149
Fatigue	1.375	0.126; 2.625	0.031
Pain	0.420	0.285; 0.555	<0.001
Swollen joint count	−3.542	−4.631; −2.453	<0.001
Clinically damaged joint	−0.017	−0.334; 0.300	0.916
Dactylitis count	−0.648	−2.638; 1.341	0.523
LEI	−1.984	−5.509; 1.541	0.270
PASI	−0.325	−0.838; 0.188	0.215
HAQ‐DI	−1.153	−7.730; 5.425	0.731
SF‐36 RP	0.096	−0.028; 0.220	0.127
SF‐36 SF	−0.013	0.088; −0.186	0.880
SF‐36 MH	−0.230	−0.407; −0.054	0.010

BMI, body mass index; LEI, Leeds Enthesitis Index; PASI, Psoriasis Area and Severity Index; HAQ‐DI, Health Assessment Questionnaire  disability index; SF‐36, Medical Outcome Short Form 36; RP, role‐physical; MH, mental health; SF, social functioning.

## 4 DISCUSSION

In this prospective study, age, fatigue, pain score, and poorer mental health positively correlated with PGA‐PhGA, while swollen joint count negatively correlated with PGA‐PhGA. To our knowledge, this is the first study to identify factors associated with discordance in an Asian population. This is also the first study to identify mental health as a factor associated with discordance in PGA and PhGA.

Discordance between patients and physicians hamper shared decision‐making, which may in turn affect long‐term outcomes. The topic of discordance between patients and physicians have been well studied in rheumatoid arthritis and axial spondyloarthritis, but only reported in a few cohorts in PsA.^11^, ^12^, ^13^ Rates of discordance were reported to be 31.2%, 56.5% and 29.1% in other PsA cohorts in Canada,^13^ Denmark^11^ and multiple European countries,^12^ respectively. Differences in discordance rates may have been related to differences in definition of discordance cut‐offs of >20/100 vs >30/100. Our discordance rate of 38.0% corroborates with the other studies to suggest that discordance is common. Discordance between physicians’ and patients’ perspectives may have important clinical implications. The discordance between change in active joint count by physicians and change in PGA was found to be a negative predictor of achieving remission, which is the target of treatment in clinical practice.^32^ In rheumatoid arthritis, discordance was also reported to be associated with reduction in work productivity.^26^, ^33^ Identifying factors associated with discordance may give insight to facilitate shared decision‐making, optimize treatment outcomes, reduce functional disability and reduce healthcare costs.

Studies have revealed that perceptions of health and illness differ in different socio‐cultural contexts,^15^, ^16^ and people from different ethnic groups differ in their perceptions and reporting of pain.^19^ We have previously shown in our PsA cohort that Indian patients had worse disease activity, pain score and PGA compared to Chinese patients.^14^ In the current study, we noted a higher proportion of non‐Chinese had positive discordance and higher proportion of Chinese had negative discordance than the concordance group; however, this was not statistically significant. In our previous study, we have also shown that the differences in disease activity between ethnic groups were mainly driven by pain score and tender joint count,^14^ and these two factors could have accounted for the majority of the variability of PGA‐PhGA rather than ethnicity per se. Another study had noted a higher discordance among women compared to men,^11^ but again the difference in proportion in discordance between genders was not statistically significant in our cohort. Despite differences in ethnicity and culture, the findings of our study conducted in a multi‐ethnic Asian setting were generally consistent with other Caucasian studies,^12^, ^13^ revealing factors influencing patient's perspective on disease activity to be more subjective (pain and fatigue) while factors affecting physician's perspective may be more objective (joint counts).

In this study, we revealed the importance of mental health in influencing discordance. Psychological factors such as mental health status and emotional well‐being play an important role in a patient's assessment of disease activity.^34^, ^35^ It has been shown that PsA patients have higher scores for anxiety and depression compared to healthy controls, and both anxiety and depression were related to pain and fatigue.^34^ Emotional well‐being was revealed as an important impact of PsA in an international qualitative study and was regarded by patients to be highly relevant in the assessment of impact of PsA.^37^ In a European study, lower self‐perceived coping and impaired social participation were found to be independent factors explaining discordance.^12^ Similarly, in our study, we found mental health as an independent risk factor for discordance. This could be an area that the physician underestimates. It is therefore an important area for physicians to focus on in the daily management of PsA.

The strength of the current study includes using validated outcomes instruments for the measurement of joint count, physical function and various PROMs, which enable valid comparison between groups. The PGA question we asked was specific to assessment of disease activity rather than general well‐being that minimizes the attributes to other comorbid conditions. Data was taken from a prospective cohort that mimics the assessment of disease activity in daily clinical practice. We controlled for several sociodemographic variables in the statistical model, such as ethnicity, educational level and BMI that may possibly influence PGA‐PhGA.

There are limitations to our study. There were several physicians assessing patients in the PsA clinic. Differences in physician characteristics could contribute to discordance.^38^ However, three physicians were designated to the care of PsA patients and standardization of scoring was performed at the beginning of the clinic start‐up. The sample size of our cohort was small, but has improved with the prospective dataset with repeated measures. There were low completion rates of protocol at all followup time points, as the cohort was built upon real‐life busy clinics where manpower to support administration of PROMs was limited. Nonetheless, PGA‐PhGA did not seem to be related to follow‐up times in our sensitivity analysis. We did not assess PGA separately for skin vs arthritis, but from a previous study,^28^ PGA should represent more arthritis than skin, particularly in a cohort with low median PASI. Moreover, we did not collect data and therefore cannot account for chronic widespread pain syndromes and fibromyalgia which are well‐known to have large effects on pain and PGA,^39^ and theoretically may have less impact on PhGA.

In conclusion, we found that age, fatigue level, pain score, number of swollen joints and mental health were associated with discordance between PGA and PhGA. Age, fatigue level, pain score, and poorer mental health were positively associated with PGA‐PhGA, while swollen joint was negatively associated with PGA‐PhGA.

## ETHICAL DISCLOSURE

Ethics approval from SingHealth CIRB Ref: 2012/498/E.

## CONFLICTS OF INTEREST

No authors declare conflicts of interest related to the production of this manuscript.
