# Supplementary material for: Factors associated with patient‐physician discordance in a prospective cohort of patients with psoriatic arthritis: An Asian perspective
Source: Int J Rheum Dis. 2019 Apr 3;22(7):1209–15. doi: 10.1111/1756-185X.13568 (PMC6766962; doi:10.1111/1756-185X.13568)
Supplement: Supplementary file 1 [file APL-22-1209-s001.docx]

**Supplementary Table 1**. Follow-up status of the prospective cohort (n=142)

|  | **Visit 1**  **Baseline** | **Visit 2**  **4 month** | **Visit 3**  **8 month** | **Visit 4**  **1 year** | **Visit 5**  **3 year** | **Visit 6**  **5 year** |
| --- | --- | --- | --- | --- | --- | --- |
| **Number of patients reaching specific time point since recruitment** | 142 | 137 | 128 | 122 | 80 | 7 |
| **Number of patients completed protocol** | 142 | 38 | 17 | 59 | 30 | 5 |
